# Unifying Inference of Meso-Scale Structures in Networks

**DOI:** 10.1371/journal.pone.0143133

**Published:** 2015-11-16

**Authors:** Birkan Tunç, Ragini Verma

**Affiliations:** Center for Biomedical Image Computing and Analytics, University of Pennsylvania, Philadelphia, Pennsylvania, United States of America; UNIVERSITY OF LAUSANNE, SWITZERLAND

## Abstract

Networks are among the most prevalent formal representations in scientific studies, employed to depict interactions between objects such as molecules, neuronal clusters, or social groups. Studies performed at meso-scale that involve grouping of objects based on their distinctive interaction patterns form one of the main lines of investigation in network science. In a social network, for instance, meso-scale structures can correspond to isolated social groupings or groups of individuals that serve as a communication core. Currently, the research on different meso-scale structures such as community and core-periphery structures has been conducted via independent approaches, which precludes the possibility of an algorithmic design that can handle multiple meso-scale structures and deciding which structure explains the observed data better. In this study, we propose a unified formulation for the algorithmic detection and analysis of different meso-scale structures. This facilitates the investigation of hybrid structures that capture the interplay between multiple meso-scale structures and statistical comparison of competing structures, all of which have been hitherto unavailable. We demonstrate the applicability of the methodology in analyzing the human brain network, by determining the dominant organizational structure (communities) of the brain, as well as its auxiliary characteristics (core-periphery).

## Introduction

At the core of any scientific pursuit stands a correspondence between objects of interest and an appropriate representation, most commonly a mathematical one. The way we represent objects in our models determines both the syntactical characteristics and semantic scope of the formalization used in the scientific modeling. Most systems of interest in social, biological, and physical sciences today consist of structurally organized objects and their interactions. Thus, many questions regarding the current and future states of such systems pertain to their architectural properties. This fact naturally explains the increasing popularity of network representations as favored in various domains of science. Networks are used to represent structured systems by referring to each object as a node and an interaction between a pair of objects as an edge between two nodes [1].

A network representation facilitates inference of system properties at various levels such as local, global (gestalt), and intermediate-scale (meso-scale) features [2]. While the overall attention directed to network analysis has increased at every level of study, studies performed at meso-scale currently form the main line of investigation. Meso-scale structures refer to grouping of nodes based on their distinctive interaction patterns. In a social network, meso-scale structures can correspond to isolated social groupings or groups of individuals that serve as a communication core [1–3]. In brain networks, the identification of meso-scale structures can reveal how the complex behavioral repertoire of the human mind emerges from the parallel processes of segregated neuronal clusters and their integration during complicated cognitive tasks [4,5].

**Fig 1:**
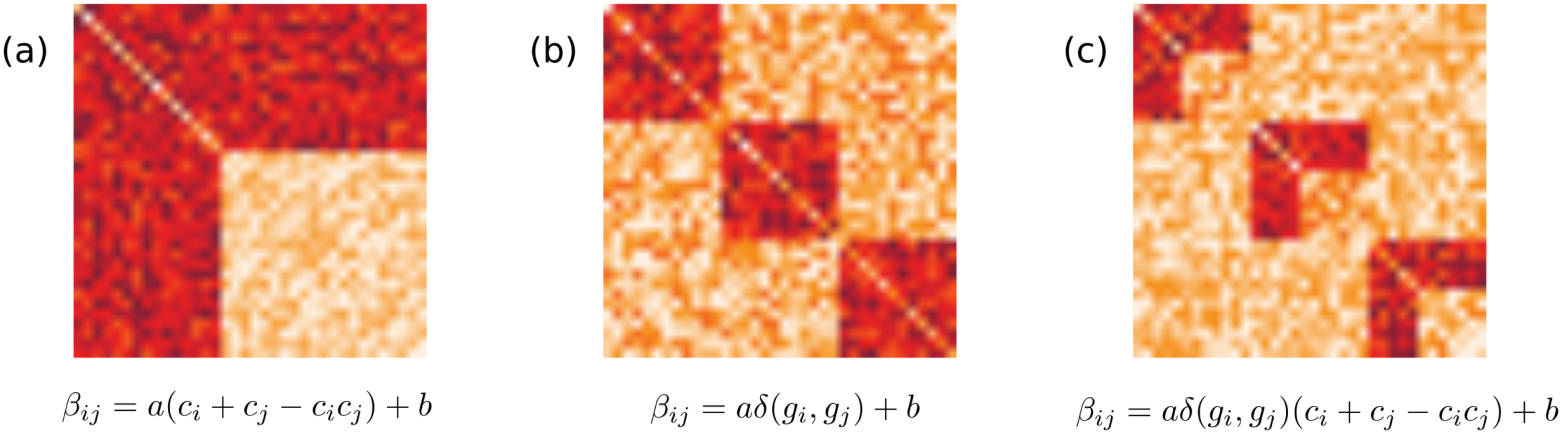
Networks with different meso-scale structures and their generative models. Networks are illustrated by their connectivity matrices depicting weights of edges between nodes. (a) A network with core-periphery structure. (b) A network with community structure. (c) A hybrid network model including both.

Several meso-scale network structures that are common to many networks, such as the community and core-periphery structures, have been identified and studied in the literature [1,2,6,7]. The effort is mostly concentrated on algorithmic detection of such special arrangements of nodes and deciding whether their presence is reliable in a statistical sense, by comparing to some null models *i*.*e*. random networks sharing several characteristics with the original network. However, the research on different meso-scale structures has been conducted by independent approaches, each employing unique methodologies and techniques. This fact precludes the possibility of an algorithmic design that can handle multiple meso-scale structures and deciding which structure explains the observed data better. For instance, although the possibility of simultaneous presence of different structures (*e*.*g*. communities of core-periphery structures, see Fig 1c for an illustration) in a network has been acknowledged [2,8], no formulation to derive such complex hierarchical structures has been proposed. Similarly, while comparison with null models has been already widely practiced [6], no work has been proposed to compare two or more competing models, each including different meso-scale structures. This is mainly due to the utilization of different formulations for different meso-scale structures.

In this study, we present a new approach that uses the Bayesian network inference framework [9] to unify the detection and analysis of meso-scale structures, thereby addressing aforementioned limitations of complex network analysis. This approach allows the identification of hybrid structures that capture the interplay between multiple meso-scale structures and the statistical comparison of competing meso-scale structures.

## Methods and Materials

### Bayesian Network Inference

A system of objects and their interactions can be represented as a network, the mathematical description of which is a graph *G* = (*V*,*E*). A graph consists of vertices (nodes) *V* = {*V*_1_,*V*_2_,…,*V*_*N*_} corresponding to objects and edges *E* ⊆ *VxV* corresponding to interactions between objects. Here, we do not assume any special type of graphs such as directed, undirected, weighted, or unweighted, since the proposed approach can be utilized for any kind. An edge between two nodes of a network indicates the existence of a relationship between corresponding objects and, when weighted, quantifies that relationship. For instance, when representing a set of time series as a network, edges can be weighted by correlations between pairs of time series. Similarly, for a social network, edges can correspond to email traffic between individuals with weights quantifying the number of email transactions.

Bayesian network inference [9,10] starts with a probabilistic treatment of observations related to the interactions between objects. The presence of edges between nodes is modelled by probability distributions over weights. In the following subsections, we elucidate the probabilistic generative model by which a network is assumed to be constructed. First, we introduce the likelihood model of observed edges between nodes. Then, we show how prior assumptions on edge formation can be incorporated into the model, which in turn enables us to infer meso-scale structures in a network.

#### Likelihood model for interactions between objects

When modeling interactions between objects, different probability distributions can be adopted, based on properties of objects and type of interactions under considerations. Following the general tendency [9,11], we assume a multinomial distribution to model the presence of edges. For an object *i*, the probability of observing a set of interactions **x**_i_ = (x_i1_,x_i2_,…,x_iN_) with *N* other objects (including itself) is modeled by a multinomial distribution

$$\text{p}\left(x_{\text{i}}|\alpha_{\text{i}}\right)=\frac{{\text{x}}_{\text{i}}!}{\prod_{\text{j}=1}^{\text{N}}{\text{x}}_{\text{ij}}!}\;\prod_{\text{j}=1}^{\text{N}}\alpha_{\text{ij}}{}^{{\text{x}}_{\text{ij}}},$$ (1)

where ${\text{x}}_{\text{i}}=\sum_{\text{j}=1}^{\text{N}}{\text{x}}_{\text{ij}}$ is the total number of observed interactions, among which x_ij_ interactions are observed between objects i and j. The parameters α_ij_ are probabilities for observing a single interaction between objects i and j, with $\sum_{\text{j}=1}^{\text{N}}\alpha_{\text{ij}}=1$. We then define a prior distribution over these probabilities using a Dirichlet distribution

$$\text{p}\left(\alpha_{\text{i}}|\beta_{\text{i}}\right)=\frac{\Gamma (\beta_{\text{i}})}{\prod_{\text{j}=1}^{\text{N}}\Gamma \left(\beta_{\text{ij}}\right)}\;\prod_{\text{j}=1}^{\text{N}}\alpha_{\text{ij}}{}^{\beta_{\text{ij}}-1},$$ (2)

where $\beta_{\text{i}}=\sum_{\text{j}=1}^{\text{N}}\beta_{\text{ij}}$ with β_ij_>0. The parameter β_ij_ can be thought as a pseudo-count for interactions i.e. how many interactions are postulated between object i and j, before making any observation. These parameters will be essential for defining meso-scale structures; therefore, we want to derive the likelihood of interactions **x**_i_ given **β**_i_, by integrating out the parameter set **α**_i_.

$$\text{p}\left(x_{\text{i}}|\beta_{\text{i}}\right)=\;\int \text{p}\left(x_{\text{i}}|\alpha_{\text{i}}\right)\text{ p}\left(\alpha_{\text{i}}|\beta_{\text{i}}\right)\text{ d}\alpha_{\text{i}}.$$

The result of this integration is the well known Dirichlet-Multinomial distribution (a.k.a Pólya distribution) [11]. Finally, the likelihood for all objects and their interactions has the following form, assuming independence between observations related to individual objects.

$$\text{p}\left(X|\text{G}\right)=\;\prod_{\text{i}=1}^{\text{N}}\left(\frac{{\text{x}}_{\text{i}}!}{\prod_{\text{j}=1}^{\text{N}}{\text{x}}_{\text{ij}}!}\;\frac{\Gamma (\beta_{\text{i}})}{\Gamma \left(\gamma_{\text{i}}\right)}\prod_{\text{j}=1}^{\text{N}}\frac{\Gamma \left(\gamma_{\text{ij}}\right)}{\Gamma \left(\beta_{\text{ij}}\right)}\right),$$ (3)

where γ_ij_ = β_ij_ + x_ij_ and $\gamma_{\text{i}}=\sum_{\text{j}=1}^{\text{N}}\gamma_{\text{ij}}$. The graph G represents our prior belief on the interactions between objects based on parameters β_ij_. In another words, G is constructed with edges having weights β_ij_.

#### Prior model for the underlying network

The total likelihood of observing a set of interactions, **X**, between objects is given by the multivariate Pólya distribution in Eq 3. The postulated structure of the underlying network is parameterized by β_ij_ that encodes our belief in possible interactions between objects i and j. Any prior assumption on the structure of the network can be incorporated by assigning appropriate values for parameters β_ij_. For instance, given a set of observations, **X**, we can estimate the underlying network structure by finding parameters **B** = {**β**_1_,**β**_2_,…,**β**_N_} that maximize the likelihood in Eq 3. Alternatively, we can further assume a prior distribution on G (equivalently on **B**) and then try to maximize the posterior distribution of G, p(G|**X**) ∝ p(**X**|G)p(G), instead of the likelihood. For both approaches, computational sampling methods such as MCMC can be used [12]. Details of such an inference scheme are available in ref. [9]. What we are interested in here is an approach to infer meso-scale structures in a network by utilizing a similar scheme, and this is explained next.

### Inference of Meso-scale Structures

In the current literature, inference of different meso-scale structures such as communities and core-periphery structures are performed by defining different maximization problems, each specific to the problem under consideration. For instance, communities are identified by maximizing the difference between the observed and expected interactions for nodes of the same communities. The modularity measure Q = ∑_i,j_(x_ij_−p_ij_)δ(g_i_, g_j_) is commonly used for this purpose, where p_ij_ is the expected number of interactions between objects i and j that are assigned to communities g_i_ and g_j_, respectively [1]. δ is the Dirac delta function having a value of 1 when g_i_, g_j_ are same and 0 otherwise. One of possible definitions for a core-periphery structure is to assume a densely connected core with objects having interactions with each other, and a periphery with objects having interactions only with the core but not with each other. With such a definition in mind, identification of the core and periphery nodes of a network is achieved by maximizing a core quality function by assigning a coreness value, c_i_, to each node. A common choice for the core quality function has the form R = ∑_i,j_x_ij_c_i_c_j_, where a transition function for coreness values is stipulated to characterize the search space for c_i_ [2]. Other type of definitions such as k-core definition[13], the definition based on the flexibility of objects in moving between communities[14], or based on the extent of integration between communities[15] would require different identification methods.

The Bayesian network inference framework introduced in the previous section enables us to unify these and similar structures. By simply answering the question, “Do we expect an interaction between objects i and j if …?” we design a generative model for the network, which in turn marks the possible values of parameter β_ij_. The antecedent of the conditional in the question can be substituted by “if they are in the same community”, “if i is a core node and j is a periphery node”, and any other form to determine the expected interactions between nodes. Three example generative models for communities, core-periphery structures, and a hybrid model including both, are introduced in the following sections. These structures were studied in this work since both the community and core-periphery structures are amongst the most studied meso-scale network structures in the literature.

#### Communities

When we have communities in a network, the ideal case is described by the condition that interactions should exist between objects of the same community, but not between objects of different communities. This can easily be simulated by the parameter β_ij_ defined as

$$\beta_{\text{ij}}=\text{ a}\delta \left({\text{g}}_{\text{i}},{\text{g}}_{\text{j}}\right)+\text{b},$$ (4)

where the scalars a and b incorporate our belief on the uncertainties of interactions (how far away we are from the ideal case). As we increase b, we increase the chance of interactions between objects of different communities. Increasing *a*, on the other hand, increases the dominance of interactions between objects of the same communities. While both scalars are informative in general applications, for the purpose of comparing different meso-scale structures, we set *a* = 1 and *b* ≈ 0 (very small number larger than zero) in all of our experiments. In the remainder of the paper, we will use the abbreviation “CS” to refer to a community structure defined by Eq 4.

#### Core-periphery structure

Similar to communities, core-periphery structures can be simulated by an appropriate choice of β_ij_. In an ideal case of core-periphery structure, we expect interactions between two core nodes and between one core node and one periphery node, whereas no interaction is expected between two periphery nodes. This expectation can be modeled by the choice

$$\beta_{\text{ij}}=\text{ a}({\text{c}}_{\text{i}}+{\text{c}}_{\text{j}}-{\text{c}}_{\text{i}}{\text{c}}_{\text{j}})+\text{b},$$ (5)

where parameters c_i_, and cj encode the coreness of nodes. Here, we assume the simplistic case of binary assignment *i*.*e*. c_i_ is either 0 (a periphery node) or 1 (a core node). Scalars a, b again govern the uncertainty similar to the case of communities. Another alternative can be defined as

$$\beta_{\text{ij}}=\text{ a}({\text{c}}_{\text{i}}+{\text{c}}_{\text{j}})+\text{b}.$$ (6)

In this case, more interactions are expected between core nodes compared to the interactions between core and periphery nodes, and again no interaction is expected between periphery nodes.

#### Hybrid models

One of the most important features of our approach is the ease of defining hybrid models. Without changing our thought process, we simply incorporate a different expectation on the interactions between objects. For a network that includes both community and core-periphery structures, our expectations can be modeled by either of the following choices.

$$\beta_{\text{ij}}=\text{ a}\delta \left({\text{g}}_{\text{i}},{\text{g}}_{\text{j}}\right)({\text{c}}_{\text{i}}+{\text{c}}_{\text{j}}-{\text{c}}_{\text{i}}{\text{c}}_{\text{j}})+\text{b},$$ (7)

$$\beta_{\text{ij}}=\text{ a}\delta \left({\text{g}}_{\text{i}},{\text{g}}_{\text{j}}\right)({\text{c}}_{\text{i}}+{\text{c}}_{\text{j}})+\text{b}.$$ (8)

These choices impose that interactions occur only when two objects are in the same community, and for objects of the same community, all node pairs except the periphery nodes have interactions. Such an explicit merger of the formulations of community and core-periphery structures is an important improvement over current approaches, as we know exactly which community each node belongs to, as well as its coreness value once the inference is done. Different model choices are illustrated in Fig 1. We used both hybrid structures that are defined by Eqs 7 and 8 in our experiments, in order to validate that the proposed Bayesian inference scheme could distinguish two core-periphery structures that have only a slight difference in their definition. They will be referred as “HS1” and “HS2” in the following sections.

#### Inferring meso-scale structures

After we decide what kind of meso-scale structure we want to detect, by assigning proper values to β_ij_, either or both of the unknown parameters c_i_, g_i_, can be inferred by maximizing the likelihood in Eq 3. When using a technique such as simulated annealing [16] for optimization, all we need is an update rule to assign new values to unknown parameters. In the case of core-periphery structure with binary assignment (*i*.*e*. 1 for core, 0 for periphery), we randomly select a node *i* and update its coreness to ${\text{c}}_{\text{i}}^{\text{new}}=1-{\text{c}}_{\text{i}}^{\text{old}}$, and probabilistically decide whether to accept this choice or not. At each iteration, parameters β_ij_ are updated based on values **c** = (c_1_,c_2_,…,c_N_) using either Eqs 5 or 6. A similar approach can be used for community detection; this time the update rule assigns a randomly chosen node *i* to a randomly decided community.

In the case of a hybrid model, the Expectation-Maximization (EM) [17] can be employed for inference. We assume that the observed network is drawn from a mixture of distributions

$$\text{p}\left(X,Z\right)=\;\prod_{\text{i}=1}^{\text{N}}\prod_{\text{k}=1}^{\text{K}}\pi_{\text{k}}^{{\text{z}}_{\text{ik}}}\text{ p}{\left(x_{\text{i}}{|\text{G}}_{\text{k}}\right)}^{{\text{z}}_{\text{ik}}},$$

where the variable z_ik_ has value of 1 if object *i* is a member of community k, and 0 if not (for each object *i*, only one z_ik_ is nonzero since we do not assume multiple memberships). The variable π_k_ is the ratio of nodes assigned to the community k. The parameters β_ij_ for a mixture component *G*_*k*_ is defined by β_ij_ = aδ(z_ik_, z_jk_)(c_i_+c_j_−c_i_c_j_)+b. Parameters **Z** and **c** are determined simultaneously, similar to the mixture of Gaussians model [18]. First, we estimate the expected value of the variable **Z** and then calculate the parameters **c** based on this expectation. This is repeated until a convergence is achieved. In the expectation step, we calculate the expected value of z_ik_ by

$$\text{E}\left[{\text{z}}_{\text{ik}}\right]=\;\frac{\pi_{\text{k}}\text{ p}\left(x_{\text{i}}{|\text{G}}_{\text{k}}\right)}{\sum_{\text{j}}\pi_{\text{j}}\text{ p}\left(x_{\text{i}}{|\text{G}}_{\text{j}}\right)}.$$

Then in the maximization step, we maximize the expectation over the log-likelihood to calculate **c**.

$$\text{E}\left[\text{lnp}\left(X,Z\right)\right]=\;\sum_{\text{i}}\sum_{\text{k}}\text{E}\left[{\text{z}}_{\text{ik}}\right]\left(\text{ln}\pi_{\text{k}}+\text{lnp}(x_{\text{i}}|{\text{G}}_{\text{k}})\right).$$

### Comparison of Competing Models

One significant asset of the proposed methodology is the fact that whole Bayesian model comparison techniques are readily applicable for comparing different network models, each assuming the presence of different meso-scale structures. For instance, we can make a decision on whether the interactions between the human brain regions are better explained by a pure community structure or by a pure core-periphery structure, as well as compare both to a hybrid model. The comparison of different models is available because the value of the likelihood (Eq 3) quantifies exactly the same thing for all models *i*.*e*. the degree of compatibility between the observed interactions in the actual network and the expected interactions imposed by the assumed meso-scale structure. A null model can be designed as a random network by assigning β_ij_ = 1 for all object pairs.

### Experiments on the Human Brain Network

The attention directed to the human brain network and its graph theoretical characteristics has increased in the last decade [19–21], leading to significant advances in computational neuroscience. Specifically, identifying meso-scale structures and following their evolution in the course of development, learning, and decision-making, has been the focus of the network studies in the neuroscience domain [22–24]. In order to demonstrate the applicability of the proposed methodology in such complex systems, we investigated the underlying network model of the human brain by comparing different candidate meso-scale structures.

Diffusion tensor imaging (DTI) [25] and probabilistic tractography [26] was used for constructing the structural network of the brain. Scans of 25 participants (males with mean age 15±1) were acquired in two epochs, on the same scanner. In the first set, DTI was acquired using a monopolar+ sequence, with repetition time (TR)/echo time (TE) = 11000/75 ms, resolution = 2x2x2 mm, collecting 30 directions with b-value = 1000 s/mm^2^ and 1 b = 0 image on a Siemens Verio 3T scanner. In the second epoch, DTI was acquired at TR/TE = 11000/76 ms using a monopolar sequence. DTI measures of FA and MD were verified not to vary between scans within the two epochs. T1-weighted (TR/TE = 1900/2.54) MRI images with resolution 0.4x0.4x0.9 mm were also acquired. The T1 image of each participant was segmented into 87 anatomical regions of interest (ROIs) of the Desikan atlas [27] using Freesurfer [28]. FSL’s probtrackx was used to perform tractography seeded from each of the 87 ROIs and going to the others [26]. A 87x87 connectivity matrix A was created for each subject, where A_ij_ = (S_ij_/S_i_)∙R_i_. In this formula, S_ij_ represents the number of fibers connecting seed region i to target j, and S_i_ represents the total number of fibers emanating from region i. The normalization by R_i_, the surface area of region i, accounts for the different sizes of the 87 ROIs. The final brain network was generated by averaging the 25 individual connectivity matrices.

## Results

### Simulation Studies

We validated the reliability of the proposed methodology in inferring meso-scale structures. To do so, networks with ground-truth meso-scale structures were simulated, and then different models were compared based on their model fit (*i*.*e*. the likelihood values). We compared three meso-scale structures, namely the pure community structure (CS) (Eq 4), the hybrid structure 1 (HS1) (Eq 7), and the hybrid structure 2 (HS2) (Eq 8). Note that when assuming a single community in a hybrid structure, we get a pure core-periphery structure (as in Eqs 5 or 6). For all simulated experiments, we used a binary core-periphery assignment *i*.*e*. a node is either core or periphery.

**Fig 2:**
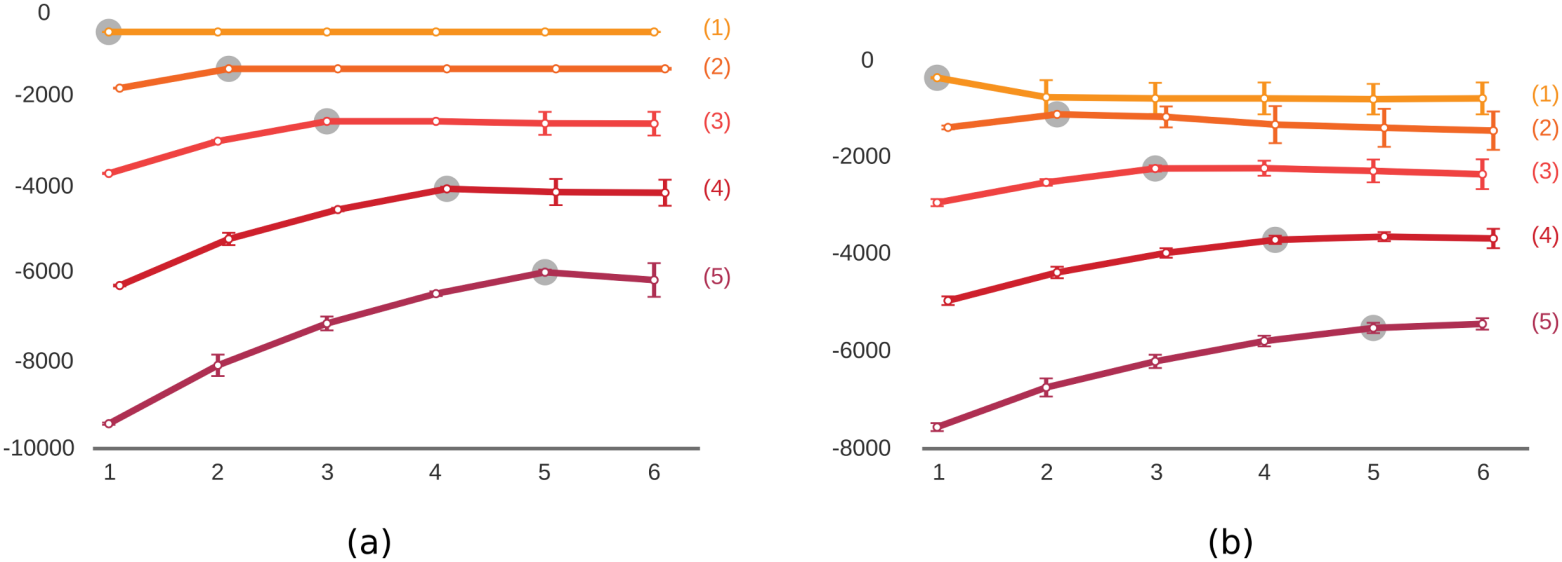
Results of simulation studies on predicting the number of communities. The true number of communities is given in parenthesis next to each curve. The proposed inference algorithm was run with different number of communities (x-axis) and the log-likelihood (y-axis) was calculated for each. The maximum log-likelihood is marked with the gray circle indicating the predicted number of communities. (a) Networks were generated using the pure community structure defined in Eq 4. (b) Networks were generated using the hybrid structure defined in Eq 7. Vertical bars show standard deviation for repeated experiments; some lines are shifted slightly along the x-axis to prevent overlaps. The true number of communities was predicted successfully for all experiments.

In the first set of experiments, we tested the capability of the method in inferring the true number of communities in the network, both for CS and the HS2. We generated networks of varying sizes and communities. For a network of m ∈ [1,5] communities, with each community including 12 nodes, the network had 12*m nodes in total. When using a hybrid structure, each community included 5 core and 7 periphery nodes. For node pairs that are expected to have interactions according to the definition of the underlying meso-scale structure, the number of interactions between them was uniformly drawn from the range [13,24]. The number of interactions between two nodes that are not expected to have any interactions was uniformly drawn from the range [0,4]. Once the network is simulated using either CS or HS2, we tried to predict the true number of communities (m), by running our proposed inference algorithm with the generative model corresponding to the true meso-scale structure and the number of communities varying in the range [1,7]. The prediction was achieved by picking the number of communities that gave the maximum value of the likelihood (Eq 3). For each value of m, this was repeated 100 times and the average was taken. Fig 2 illustrates results of these experiments, with the true number of communities being predicted accurately each time.

**Fig 3:**
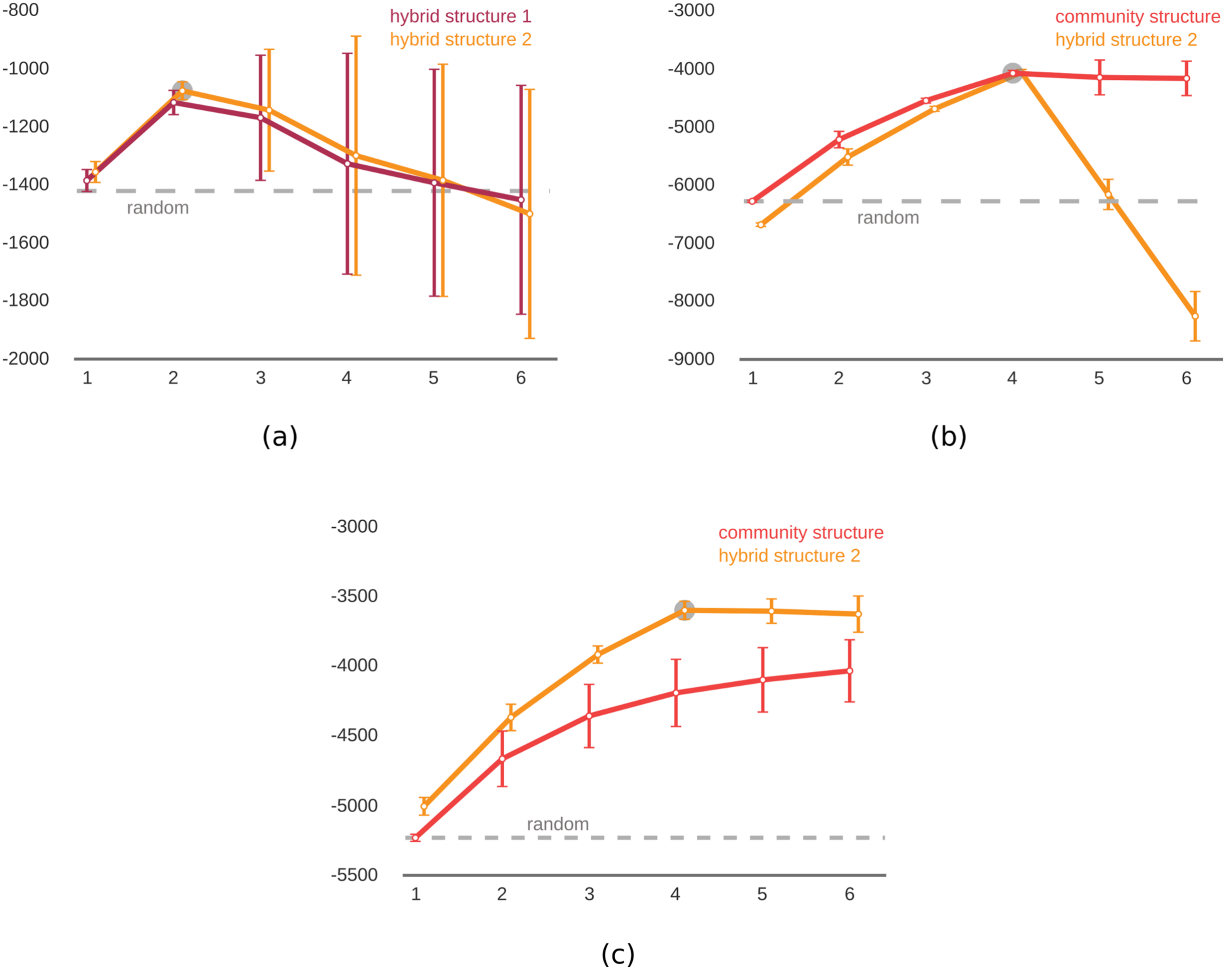
Model comparisons with different ground-truth meso-scale network structures. The proposed inference algorithm was run with different number of communities (x-axis) and the log-likelihood (y-axis) was calculated for each. The maximum log-likelihood is marked with the gray circle indicating the predicted number of communities. Dashed gray line shows the log-likelihood for a random network. The ground-truth meso-scale structure was (a) the hybrid structure 2 (Eq 8), (b) the community structure (Eq 4), (c) the hybrid structure 2 (Eq 8). Vertical bars show standard deviation for repeated experiments; some lines are shifted slightly along the x-axis to prevent overlaps. The true models achieved higher likelihood in all pairwise comparisons, with the true number of communities achieving the maximum value in each case.

With a second set of experiments, we tried to predict the true underlying meso-scale structure, *i*.*e*. CS, HS1, or HS2. Using the same test configuration of the first experiment, we first simulated random networks with selected ground-truth meso-scale structures and then tried to fit the data using all three candidates. We expected that the highest value of the likelihood would be achieved when the true number of communities and the true meso-scale structure were used. Both HS1 and HS2 consist of communities of core-periphery structures with slight differences in the core-periphery model (compare Eqs 7 and 8); therefore, distinguishing them is a hard task even for simulated networks. Fig 3a shows results when the network is simulated with the ground-truth meso-scale structure HS2 and 2 communities (similar results were produced for other number of communities in the range [1–5]). Comparisons between HS1 and HS2 are given. Both models explained the data better than a random network, with the true model (HS2) achieving higher likelihood values consistently.

Similar results are presented in Fig 3b and 3c for the cases when the ground-truth structure is CS with 4 communities and HS2 with 4 communities, respectively. Again, the true underlying model was predicted successfully each time. Note that, when the true underlying model is CS, both HS1 and HS2 can also be fitted accurately by simply assigning all nodes as core nodes (see Fig 3b). With CS, even when we try to fit the model with a number of communities that is higher than the true number, optimization may end up with the extra communities being empty, which results in the same likelihood value with the true model (see Fig 3b and 3c). This is usually not true for HS1 and HS2 due to increased number of free parameters. Overall, results in Fig 3 show that the proposed meso-scale inference methodology is sensitive to even small changes in the underlying ground-truth network structure (*e*.*g*. HS1 vs HS2), which renders our approach very reliable in model comparisons.

### The Human Brain Network

Two meso-scale structures, namely community structure (CS) and a hybrid structure fusing community and core-periphery structures (HS1) were compared. Both candidates were fitted with changing number of communities. When the number of communities is 1 for the hybrid structure, this corresponds to a pure core-periphery structure. Similar to previous experiments, a binary version of the core-periphery structure (*i*.*e*. a node is either core or periphery) was used, and the best model was selected based on the highest likelihood.

**Fig 4:**
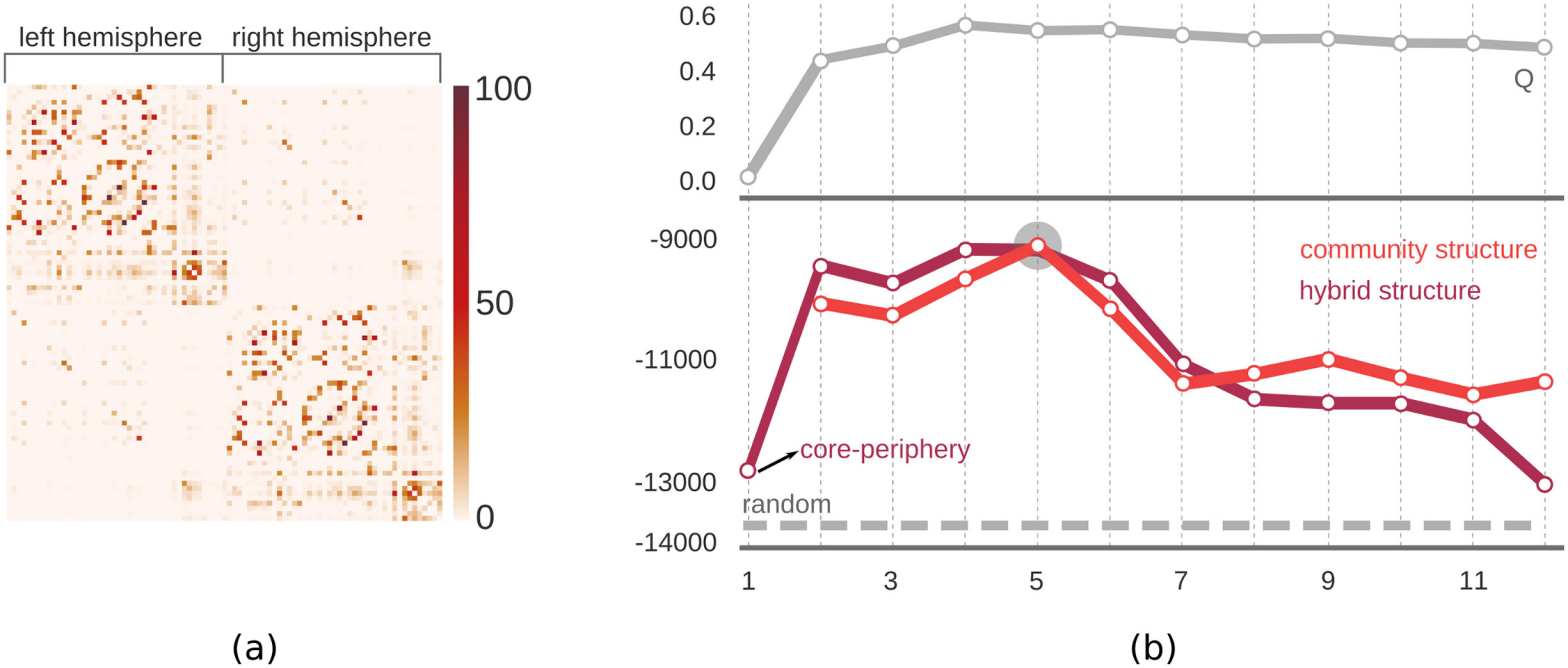
Meso-scale structures of the human brain network. (a) The connectivity matrix of the brain that defined the network. Edges between nodes were weighted by the number of streamlines (normalized so as to have values between 0 and 100). (b) Model fits with different candidate meso-scale structures; three structures were compared. The proposed inference algorithm was run with different number of communities (x-axis) and the log-likelihood (y-axis) was calculated for each. The upper panel gives the modularity measure (Q) for different number of communities. Comparison with Q shows that the change in the likelihood value as we increase the number of communities, is similar to the change in the traditionally used modularity measure.

Our methodology enables us to make very detailed interpretations on the meso-scale structure of the network. For instance, we see in Fig 4b that both models explained the observed data (the brain network) better than a random network did. The best fit corresponding to the highest likelihood was achieved by a community structure with 5 communities. The hybrid structure with a single community (i.e. a pure core-periphery structure, see the black arrow in the figure) achieved only slightly better likelihood than a random network, whereas introducing even two communities (corresponding to the left and right hemispheres of the brain), improved the model fit noticeably. The overall difference between the likelihoods of the pure community structure and the hybrid structure was minimal, suggesting that the connections between anatomical regions were mainly characterized by a community structure, but not by a core-periphery structure.

**Fig 5:**
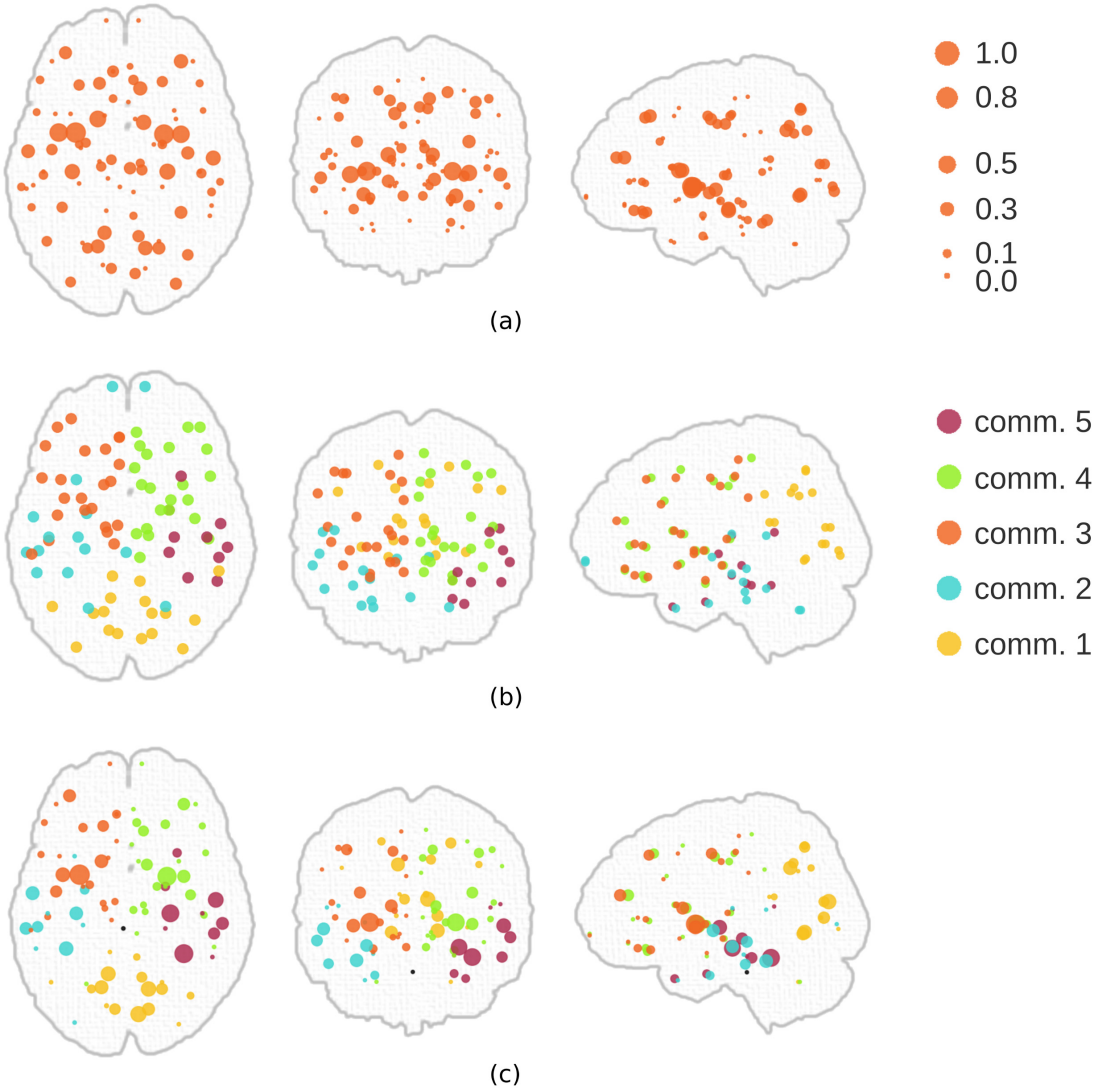
Community and core-periphery structures of the human brain network. (a) Distribution of coreness among nodes when a pure core-periphery structure is assumed. (b) Communities of the network with 5 communities. (c) A hybrid structure. The hybrid model integrates the decisions from two distinct models.

The inferred community and core-periphery structures of the human brain network are depicted in Fig 5. In order to see the distribution of coreness among nodes, we ran the proposed algorithm with a continuous coreness value, instead of a binary one. Even though the hybrid model did not contribute much in terms of explaining the data (see Fig 4b); when we compare Fig 5b with Fig 5c, we see that the assignment of nodes to communities became more intuitive (*e*.*g*. communities do not span both hemispheres) when coreness of nodes was incorporated into the hybrid model (observe the change in community #2 in Fig 5b and 5c). Such an extensive interpretation has not been hitherto available, since it is not possible to compare multiple models with the current methods of meso-scale detection, as they are identified by independent methodologies.

## Discussion

We have proposed a unified approach for identification of meso-scale network structures, such as community structures and core-periphery structures. In the current literature, such tasks are performed by utilizing methods and algorithms that are highly specific to a single structure, hindering comparison of findings. Defining a common methodology for multiple meso-scale structures is an important contribution for several reasons.

First, a common formulation of different structures naturally provides a way to define hybrid network models that combine multiple meso-scale structures with complex hierarchies. In the literature, it is a well-appreciated fact that networks corresponding to real life objects and interactions do not include a single meso-scale structure, but instead are characterized by architectures that demonstrate interplay of multiple structures. Especially for the biological networks with a high level of complexity in their functional outputs, such hybrid models are expected to be prevalent. The proposed unification facilitates an easy way to infer hybrid models, as well as very complex meso-scale structures.

Second, the proposed unification enables the comparison of competing models (see Figs 3 and 4), a subject that for the most part remains largely unexplored in the current literature. Use of null models has been very popular to quantify the reliability of the inference of the meso-scale structures in networks. Identified meso-scale structures are compared to random networks, and this requires a specific null model for each separate meso-scale structure. In the proposed methodology, null models are parameterized in the same way that meso-scale structures are parameterized. Moreover, not only the comparison to random networks, but also the comparison between different meso-scale structures is now enabled. This introduces a unique advantage in interpreting the architecture of the networks. Through our experiments on the human brain network (Figs 4 and 5), we demonstrated how this unique feature could facilitate the inference of the dominant organizational structure (communities) of the brain, as well as its auxiliary characteristics (core-periphery). It is already known that both the community structure and the core-periphery structure may exist in the human brain network [29,30]. To the best of our knowledge, for the first time, we have shown that the main governing structure is the community structure while the core-periphery structure contributes only minimally towards explaining the observed interactions among cortical regions (Fig 4b). Such a comparison was due to the common quality measure (*i*.*e*. likelihood of the model) that quantifies exactly the same thing for different models.

Third, the identification of meso-scale structures is unified using a common generative model for any kind of meso-scale structure. This was achieved by introducing a link between the formation of a network and the expectations introduced by the meso-scale structure being studied. Each meso-scale structure introduces a different set of expectations on the interactions between objects. For instance, for a community structure, we expect that the interactions occur only between the objects of the same communities. Similarly, a core periphery structure is defined by the set of expectations that decide the interactions between core nodes, between core and periphery nodes, and between periphery nodes. Since we define each meso-scale structure using the same language *i*.*e*. language of expectations, both interpretations of the formation of such structures and that of our statistical findings are now commensurable. Using this language, we can easily study more meso-scale structures such as onions [31], bow-ties [32], or other block models [33]. We can do that simply by assigning appropriate values to the parameter β_ij_ (Eq 3), which reflects the expected amount of interaction between objects when the meso-scale structure is present in the network.

It should be noted that using the raw likelihood values for model comparison is not the best approach in general. In real-world large networks, differentiating candidate models or number of communities may be difficult and unstable due to very small differences between likelihood values (see Fig 4b). This is expected when comparing two community structures with similar number of communities since difference between two candidate models can be the addition of an extra community, with only a few or sometimes no assignment (empty community) of nodes to the new community. This results in very close or equal likelihood values. Similarly, when we blend a core-periphery structure into the model to have a hybrid model, difference between the hybrid model and a pure community structure can be minimal when most of the nodes (or even all of them) are assigned as core nodes in the hybrid model, which renders it as a pure community structure. We observed such limitations in our experiments with other real-world networks, as well. It is always the best practice to inspect resulting meso-scale structure qualitatively (*e*.*g*. investigations on the assignment of nodes to communities) in addition to the quantitative analysis performed using likelihood comparison. This limitation is also evident with the traditionally used quality measures like modularity (Q). However, the proposed methodology can be used in combination with any Bayesian model selection procedure. We recommend using more sophisticated measures such as BIC [34] or AIC [35] that incorporates the model complexity into the decision process in order to reach more robust decisions. In this work, we have used the simplest measure to better demonstrate the capacity and the limitations of the proposed methodology, without embarking on a comparison of the decision measures that would detract from the focus of this work.

Much work remains to be done in future studies, pertaining both to the theoretical and practical aspects of the proposed methodology. From a theoretical perspective, advanced optimization schemes should be explored to identify optimal solutions especially for the complex hybrid models. As we introduce intricate hierarchies to capture the interplay between multiple meso-scale structures, the generative model becomes more and more underdetermined by the observations, due to an excessive number of unknown parameters. Informed prior assumptions on the network topology and geometry can be incorporated to address such concerns. Resulting complicated models can be studied by more sophisticated inference schemes such as probabilistic graphical models that break complex models into conditionally independent simpler sub-models [36]. In theory, the proposed methodology is applicable to the inference of meso-scale structures other than the community and core-periphery structures. This fact should also be validated with practical applications, to establish the effective representational capacity of our methodology. The current generative model that is mainly parameterized by β_ij_ (see Eq 3), can possibly be augmented to represent more complex meso-scale structures. We believe that the possibilities that are brought forward by the proposed unification of the inference of meso-scale network structures are critical to advances in several domains including biological, physical, and social sciences. And, these possibilities are expected to multiply with the abovementioned future improvements.
